# Supplementary material for: Regime shift detection and neurocomputational substrates for under and overreactions to change
Source: eLife. 2026 May 11;14:RP104684. doi: 10.7554/eLife.104684 (PMC13160555; doi:10.7554/eLife.104684)
Supplement: Supplementary file 1. — Cluster-level inference using Gaussian random field theory (familywise error corrected at p < 0.05 with a cluster-forming threshold z>3.1\begin{document}$z{> }3.1$\end{document}). [file elife-104684-supp1.docx]

| **Probability estimates** $\boldsymbol{P}_{\boldsymbol{t}}$ **(negative correlation)** | | | | |
| --- | --- | --- | --- | --- |
| **Cluster** | **Hemisphere** | **Cluster size** | **z-max** | **z-max(x,y,z)** |
| Central Opercular Cortex | R | 26990 | 6.13 | (62,-6,6) |
| (Local maxima) |  |  |  |  |
| Frontal Orbital Cortex |  |  | 5.80 | (−28, 32, −14) |
| Insular Cortex |  |  | 5.72 | (36, −14, 16) |
| Planum Polare |  |  | 5.58 | (−48, −8, −10) |
| Central Opercular Cortex |  |  | 5.53 | (50, −10, 12) |
| Frontal Orbital Cortex |  |  | 5.45 | (30, 32, -16) |
| Cingulate Gyrus, anterior division | **-** | 989 | 4.51 | (0,18,36) |
| (Local maxima) |  |  |  |  |
| Cingulate Gyrus, posterior division |  |  | 3.94 | (14, −34, 46) |
| Cingulate Gyrus, anterior division |  |  | 3.86 | (−8, −10, 40) |
| Cingulate Gyrus, anterior division |  |  | 3.86 | (6, 0, 40) |
| Supplementary Motor Cortex |  |  | 3.84 | (6, −8, 48) |
| Supplementary Motor Cortex |  |  | 3.81 | (6, −4, 48) |
| **Belief revision** $\boldsymbol{\Delta P}_{\boldsymbol{t}}$ **(positive correlation)** | | | | |
| Frontal Orbital Cortex | L | 3105 | 4.94 | (-24,18,-10) |
| Cingulate Gyrus, anterior division | L | 2589 | 4.34 | (-4,26,30) |
| Frontal Orbital Cortex | R | 1796 | 4.57 | (26,20,-12) |
| Right Cerebral White Matter | R | 1557 | 4.67 | (12,-14,-4) |
| Lateral Occipital Cortex, superior division | L | 1366 | 4.34 | (-28,-82,34) |
| Postcentral Gyrus | R | 1262 | 4.71 | (60,-18,36) |
| Postcentral Gyrus | L | 1088 | 4.48 | (-58,-22,36) |
| Postcentral Gyrus | L | 587 | 4.25 | (-34,-26,68) |
