# Supplementary material for: Regime shift detection and neurocomputational substrates for under and overreactions to change
Source: eLife. 2026 May 11;14:RP104684. doi: 10.7554/eLife.104684 (PMC13160555; doi:10.7554/eLife.104684)
Supplement: Supplementary file 2. — Permutation tests based on threshold-free-cluster-enhancement (TFCE) statistic. [file elife-104684-supp2.docx]

| **Probability estimates** $\boldsymbol{P}_{\boldsymbol{t}}$ **(negative correlation)** | | | | |
| --- | --- | --- | --- | --- |
| **Cluster** | **Hemisphere** | **Cluster size** | $\boldsymbol{p}_{\boldsymbol{max}}$ | **1-p_max_(x,y,z)** |
| Occipital Pole | R | 62107 | 0 | (18,-98,22) |
| Frontal Pole | L | 676 | 0.036 | (-32,40,34) |
| **Belief revision** $\boldsymbol{\Delta P}_{\boldsymbol{t}}$ **(positive correlation)** | | | | |
| Frontal Orbital Cortex | L | 75881 | 0.002 | (-22,14,-16) |
| Frontal Pole | R | 424 | 0.045 | (34,36,32) |
| Supramarginal Gyrus, posterior division | L | 53 | 0.048 | (-68,-46,10) |
