# Supplementary material for: Regime shift detection and neurocomputational substrates for under and overreactions to change
Source: eLife. 2026 May 11;14:RP104684. doi: 10.7554/eLife.104684 (PMC13160555; doi:10.7554/eLife.104684)
Supplement: Supplementary file 3. — Permutation tests based on cluster extent. [file elife-104684-supp3.docx]

| **Probability estimates** $\boldsymbol{P}_{\boldsymbol{t}}$ **(negative correlation)** | | | | |
| --- | --- | --- | --- | --- |
| **Cluster** | **Hemisphere** | **Cluster size** | $\boldsymbol{p}_{\boldsymbol{max}}$ | **1-p_max_(x,y,z)** |
| Lateral Occipital Cortex, inferior division | R | 5326 | 0 | (56,-66,-12) |
| Temporal Fusiform Cortex, anterior division | L | 1989 | 0.001 | (-38,-8,-32) |
| Planum Polare | R | 926 | 0.003 | (52,6,-4) |
| Middle Temporal Gyrus, anterior division | L | 777 | 0.004 | (-52,-4,-18) |
| Postcentral Gyrus | R | 570 | 0.006 | (44,-18,36) |
| Right Cerebral White Matter | R | 394 | 0.009 | (36,0,-30) |
| Cingulate Gyrus, anterior division | R | 156 | 0.027 | (2,30,16) |
| Middle Temporal Gyrus, posterior division | L | 89 | 0.044 | (-62,-36,0) |
| Precuneus Cortex | - | 80 | 0.048 | (0,-56,56) |
| **Belief revision** $\boldsymbol{\Delta P}_{\boldsymbol{t}}$ **(positive correlation)** | | | | |
| Cingulate Gyrus, anterior division | L | 3082 | 0.002 | (-4,44,4) |
| Frontal Orbital Cortex | L | 2083 | 0.002 | (-24,14,-26) |
| Lingual Gyrus | R | 1274 | 0.004 | (2,-78,0) |
| Frontal Orbital Cortex | R | 708 | 0.008 | (14,12,-18) |
| Planum Polare | R | 582 | 0.01 | (44,-2,-20) |
| Brain-Stem | L | 566 | 0.01 | (-4,-36,-28) |
| Postcentral Gyrus | L | 390 | 0.014 | (-58,-20,24) |
| Supramarginal Gyrus, anterior division | R | 366 | 0.014 | (70,-20,24) |
| Lateral Occipital Cortex, superior division | L | 233 | 0.024 | (-32,-78,30) |
| Precentral Gyrus | L | 207 | 0.028 | (-28,-8,54) |
| Precentral Gyrus | L | 188 | 0.03 | (-54,6,24) |
| Precentral Gyrus | R | 168 | 0.035 | (56,8,24) |
