# Supplementary material for: Regime shift detection and neurocomputational substrates for under and overreactions to change
Source: eLife. 2026 May 11;14:RP104684. doi: 10.7554/eLife.104684 (PMC13160555; doi:10.7554/eLife.104684)
Supplement: Supplementary file 4. — For Experiment 3, INt\begin{document}$\mathrm{IN}_{t}$\end{document} represents the two-digit number subjects were instructed to press at each period, and ΔINt\begin{document}$\Delta IN_{t}$\end{document} represents the difference in number between successive periods. INt\begin{document}$\mathrm{IN}_{t}$\end{document} is the control for Pt\begin{document}$P_{t}$\end{document} in Experiment 1, and ΔINt\begin{document}$\Delta IN_{t}$\end{document} is the control for ∆Pt\begin{document}$\Delta P_{t}$\end{document} in Experiment 1. Cluster-level inference using Gaussian random field theory (familywise error corrected at p < 0.05 with a cluster-forming threshold z>3.1\begin{document}$z{> }3.1$\end{document}). [file elife-104684-supp4.docx]

| **Instructed number** $\boldsymbol{IN}_{\boldsymbol{t}}$ **(negative correlation)** | | | | |
| --- | --- | --- | --- | --- |
| **Cluster** | **Hemisphere** | **Cluster size** | **z-max** | **z-max(x,y,z)** |
| Precentral Gyrus | R | 1320 | 4.66 | (38,-22,62) |
| Postcentral Gyrus | L | 521 | 4.08 | (-40,-36,62) |
| Brain-Stem | L | 419 | 4.22 | (-4,-28,-10) |
| Cerebellar Left V | L | 401 | 4.8 | (-16,-50,-20) |
| Middle Frontal Gyrus | L | 316 | 4.22 | (-40,36,32) |
| Precentral Gyrus | R | 294 | 3.95 | (56,2,38) |
| Right Thalamus | R | 248 | 4.31 | (8,-18,0) |
| Superior Frontal Gyrus | L | 223 | 3.87 | (-20,-8,70) |
| **Difference in instructed number** $\boldsymbol{\Delta IN}_{\boldsymbol{t}}$ **(positive correlation)** | | | | |
| Precentral Gyrus | L | 1489 | 5.09 | (-40,-20,54) |
