# Supplementary material for: Regime shift detection and neurocomputational substrates for under and overreactions to change
Source: eLife. 2026 May 11;14:RP104684. doi: 10.7554/eLife.104684 (PMC13160555; doi:10.7554/eLife.104684)
Supplement: Supplementary file 5. — Cluster-level inference using Gaussian random field theory (familywise error corrected at p < 0.05 with a cluster-forming threshold z>3.1\begin{document}$z{> }3.1$\end{document}). [file elife-104684-supp5.docx]

| **Experiment 1** $\boldsymbol{>}$ **Experiment 2 on negative probability estimates contrast** | | | | |
| --- | --- | --- | --- | --- |
| **Cluster** | **Hemisphere** | **Cluster size** | **z-max** | **z-max(x,y,z)** |
| Paracingulate Gyrus | L | 7726 | 4.93 | (-8,38,-8) |
| Lateral Occipital Cortex, superior division | L | 3457 | 4.95 | (-16,-86,46) |
| Postcentral Gyrus | R | 328 | 3.82 | (48,-20,64) |
