# Supplementary material for: Regime shift detection and neurocomputational substrates for under and overreactions to change
Source: eLife. 2026 May 11;14:RP104684. doi: 10.7554/eLife.104684 (PMC13160555; doi:10.7554/eLife.104684)
Supplement: Supplementary file 6. — Cluster-level inference using Gaussian random field theory (familywise error corrected at p<0.05 with a cluster-forming threshold z>3.1). [file elife-104684-supp6.docx]

| **Experiment 1** $\boldsymbol{>}$ **Experiment 2 on negative probability estimates contrast** | | | | |
| --- | --- | --- | --- | --- |
| **Cluster** | **Hemisphere** | **Cluster size** | **z-max** | **z-max(x,y,z)** |
| Superior Temporal Gyrus, anterior division | L | 4099 | 5.04 | (-60,2,-2) |
| Occipital Pole | R | 2656 | 5.21 | (16,-98,28) |
| Central Opercular Cortex | R | 1575 | 5.41 | (62,-6,8) |
| **Experiment 1** $\boldsymbol{>}$ **Experiment 2 on positive probability estimates contrast** | | | | |
| Supramarginal Gyrus, anterior division | L | 328 | 4 | (-52,-38,44) |
