# Supplementary material for: Regime shift detection and neurocomputational substrates for under and overreactions to change
Source: eLife. 2026 May 11;14:RP104684. doi: 10.7554/eLife.104684 (PMC13160555; doi:10.7554/eLife.104684)
Supplement: Supplementary file 7. — Cluster-level inference using Gaussian random field theory (familywise error corrected at p < 0.05 with a cluster-forming threshold z>3.1\begin{document}$z{> }3.1$\end{document}). [file elife-104684-supp7.docx]

| **Probability estimates** $\boldsymbol{P}_{\boldsymbol{t}}$ **(negative correlation)** | | | | |
| --- | --- | --- | --- | --- |
| **Cluster** | **Hemisphere** | **Cluster size** | **z-max** | $\boldsymbol{z-}\mathbf{max}\boldsymbol{(x,y,z)}$ |
| Lingual Gyrus | L | 11504 | 6.2 | (-14,-52,-12) |
| (Local maxima) |  |  |  |  |
| Temporal fusiform cortex, posterior division |  |  | 5.12 | (−20, −46, −24) |
| Left accumbens |  |  | 5.11 | (−4, 8, −4) |
| Occipital pole |  |  | 5.11 | (16, −98, 28) |
| Superior temporal Gyrus |  |  | 5.10 | (−54, −4, −6) |
| Occipital pole |  |  | 5.06 | (12, −88, 40) |
| Central Opercular Cortex | R | 9872 | 6.19 | (60,-8,6) |
| (Local maxima) |  |  |  |  |
| Central Opercular Cortex |  |  | 6.13 | (56, −8, 10) |
| Central Opercular Cortex |  |  | 6.11 | (62, −4, 8) |
| Insular Cortex |  |  | 5.85 | (34, −14, 14) |
| Postcentral Gyrus |  |  | 5.85 | (44, −28, 64) |
| Precentral Gyrus |  |  | 5.54 | (40, −24, 58) |
| **Probability estimates (positive correlation)** | | | | |
| Postcentral Gyrus | L | 1680 | 5.45 | (-46,-26,56) |
| **Belief revision** $\boldsymbol{\Delta P}_{\boldsymbol{t}}$ **(positive correlation)** | | | | |
| Left Cerebral White Matter | L | 717 | 4.33 | (-20,18,-12) |
| Cingulate Gyrus, anterior division | - | 566 | 4.39 | (0,32,-8) |
| Frontal Orbital Cortex | R | 475 | 4.17 | (20,8,-16) |
| Postcentral Gyrus | L | 333 | 4.02 | (-30,-28,64) |
| **Intertemporal prior (negative correlation)** | | | | |
| Occipital Fusiform Gyrus | R | 1833 | 4.61 | (36,-74,-16) |
| Lateral Occipital Corte | R | 223 | 4.04 | (36,-66,-56) |
| Lateral Occipital Cortex | L | 204 | 4.25 | (-40,-82,-8) |
| $\mathbf{ln}\left( \boldsymbol{d} \right)$**× signal (positive correlation)** | | | | |
| Middle Frontal Gyrus | R | 988 | 4.17 | (36,10,30) |
| Superior Frontal Gyrus | R | 821 | 4.56 | (6,34,48) |
| Superior Parietal Lobule | L | 620 | 4.26 | (-40,-52,56) |
| Supramarginal Gyrus | R | 604 | 4.29 | (46,-40,48) |
| Middle Frontal Gyrus | L | 277 | 4.21 | (-46,34,30) |
