# Supplementary material for: Regime shift detection and neurocomputational substrates for under and overreactions to change
Source: eLife. 2026 May 11;14:RP104684. doi: 10.7554/eLife.104684 (PMC13160555; doi:10.7554/eLife.104684)
Supplement: Supplementary file 8. — Permutation tests based on the threshold-free-cluster-enhancement (TFCE) statistic. [file elife-104684-supp8.docx]

| **Probability estimates** $\boldsymbol{P}_{\boldsymbol{t}}$ **(negative correlation)** | | | | |
| --- | --- | --- | --- | --- |
| **Cluster** | **Hemisphere** | **Cluster size** | $\boldsymbol{p}_{\boldsymbol{max}}$ | $\boldsymbol{1-}\boldsymbol{p}_{\boldsymbol{max}}\boldsymbol{(x,y,z)}$ |
| Postcentral Gyrus | R | 31490 | 0 | (38,-24,50) |
| Frontal Pole | R | 165 | 0.038 | (16,36,-20) |
| **Probability estimates** $\boldsymbol{P}_{\boldsymbol{t}}$ **(positive correlation)** | | | | |
| Postcentral Gyrus | L | 439 | 0.012 | (-42,-28,54) |
| **Belief revision** $\boldsymbol{\Delta P}_{\boldsymbol{t}}$ **(positive correlation)** | | | | |
| Right Caudate | R | 2857 | 0.027 | (12,14,-4) |
| Cingulate Gyrus, anterior division | R | 1873 | 0.027 | (4,26,24) |
| Postcentral Gyrus | L | 707 | 0.034 | (-42,-22,54) |
| Insular Cortex | L | 427 | 0.041 | (-42,6,-4) |
| Frontal Medial Cortex | L | 320 | 0.04 | (-4,40,-14) |
| Frontal Pole | L | 40 | 0.048 | (-2,60,28) |
| Insular Cortex | R | 20 | 0.048 | (40,6,-14) |
| **Intertemporal prior (negative correlation)** | | | | |
| Lateral Occipital Cortex, inferior division | R | 2117 | 0.006 | (38,-74,14) |
| Frontal Pole | R | 185 | 0.038 | (36,42,36) |
| Frontal Pole | R | 114 | 0.043 | (40,44,-6) |
| Middle Frontal Gyrus | R | 14 | 0.048 | (34,28,50) |
| Right Thalamus | R | 13 | 0.046 | (22,-28,-2) |
| Frontal Pole | R | 1 | 0.05 | (32,48,-8) |
| Frontal Pole | R | 1 | 0.05 | (30,52,-6) |
