# Supplementary material for: Regime shift detection and neurocomputational substrates for under and overreactions to change
Source: eLife. 2026 May 11;14:RP104684. doi: 10.7554/eLife.104684 (PMC13160555; doi:10.7554/eLife.104684)
Supplement: Supplementary file 9. — Permutation tests based on cluster extent. [file elife-104684-supp9.docx]

| **Probability estimates** $\boldsymbol{P}_{\boldsymbol{t}}$ **(negative correlation)** | | | | |
| --- | --- | --- | --- | --- |
| **Cluster** | **Hemisphere** | **Cluster size** | $\boldsymbol{p}_{\boldsymbol{max}}$ | $\boldsymbol{1-}\boldsymbol{p}_{\boldsymbol{max}}\boldsymbol{(x,y,z)}$ |
| Postcentral Gyrus | R | 1770 | 0.001 | (42,-20,38) |
| Lateral Occipital Cortex, inferior division | R | 1085 | 0.003 | (60,-68,-6) |
| Lateral Occipital Cortex, inferior division | L | 674 | 0.006 | (-42,-84,10) |
| Temporal Fusiform Cortex, posterior division | L | 597 | 0.007 | (-36,-18,-32) |
| Heschl's Gyrus | R | 527 | 0.008 | (54,-12,2) |
| Temporal Fusiform Cortex, anterior division | R | 441 | 0.01 | (34,-2,-30) |
| Cerebellar Left V | L | 235 | 0.022 | (-16,-50,-24) |
| Middle Temporal Gyrus, anterior division | L | 193 | 0.027 | (-52,-4-18) |
| Frontal Medial Cortex | L | 134 | 0.039 | (-10,34,-18) |
| Lingual Gyrus | L | 134 | 0.039 | (-6,-48,-2) |
| **Probability estimates** $\boldsymbol{P}_{\boldsymbol{t}}$ **(positive correlation)** | | | | |
| Supramarginal Gyrus, anterior division | L | 1012 | 0.002 | (-44,-30,40) |
| **Belief revision** $\boldsymbol{\Delta P}_{\boldsymbol{t}}$ **(positive correlation)** | | | | |
| Cingulate Gyrus, anterior division | - | 450 | 0.016 | (0,34,14) |
| Frontal Orbital Cortex | R | 351 | 0.021 | (16,12,-16) |
| Frontal Orbital Cortex | L | 300 | 0.024 | (-22,10,-18) |
| Postcentral Gyrus | L | 215 | 0.035 | (-38,-24,46) |
| **Intertemporal prior (negative correlation)** | | | | |
| Temporal Occipital Fusiform Cortex | R | 1687 | 0.002 | (34,-60,24) |
| Lateral Occipital Cortex, superior division | R | 220 | 0.042 | (38,-70,32) |
| $\mathbf{ln}\left( \boldsymbol{d} \right)$**× signal (positive correlation)** | | | | |
| Superior Parietal Lobule | L | 384 | 0.022 | (-38,-52,40) |
