# Supplementary material for: Regime shift detection and neurocomputational substrates for under and overreactions to change
Source: eLife. 2026 May 11;14:RP104684. doi: 10.7554/eLife.104684 (PMC13160555; doi:10.7554/eLife.104684)
Supplement: Supplementary file 10. [file elife-104684-supp10.docx]

| **Model** | **Likelihood** | **Number of Parameters** | **AIC** |
| --- | --- | --- | --- |
| SN-original | $356.27 \pm44.7$ | $6$ | $-700.54 \pm89.49$ |
| SN-SigDep-$\beta$ | $383.08 \pm44.69$ | $9$ | $-748.15 \pm89.37$ |
| SN-SigDep-$\alpha$ | $398.61 \pm45.78$ | $9$ | $-779.21 \pm91.57$ |
| SN-SigDep-$\alpha\beta$ | $410.03 \pm46.37$ | $12$ | $-796.07 \pm92.74$ |
