# Supplementary material for: Regime shift detection and neurocomputational substrates for under and overreactions to change
Source: eLife. 2026 May 11;14:RP104684. doi: 10.7554/eLife.104684 (PMC13160555; doi:10.7554/eLife.104684)
Supplement: Supplementary file 11. [file elife-104684-supp11.docx]

| **Model** | $\boldsymbol{t}$ **test**  $\boldsymbol{(df=29)}$ | $\boldsymbol{p}$**-value** | **Bonferroni-corrected** $\boldsymbol{p}$**-value** |
| --- | --- | --- | --- |
| SN-SigDep-$\beta$ $-$ SN-original | $5.15$ | $0.0000$ | $0.0001$ |
| SN-SigDep-$\alpha$ $-$ SN-original | $6.93$ | $0.0000$ | $0.0000$ |
| SN-SigDep-$\alpha\beta$ $-$ SN-original | $8.56$ | $0.0000$ | $0.0000$ |
| SN-SigDep-$\alpha$ $-$ SN-SigDep-$\beta$ | $4.01$ | $0.0004$ | $0.0023$ |
| SN-SigDep-$\alpha\beta$ $-$ SN-SigDep-$\beta$ | $6.55$ | $0.0000$ | $0.0000$ |
| SN-SigDep-$\alpha\beta$ $-$ SN-SigDep-$\alpha$ | $5.03$ | $0.0000$ | $0.0001$ |
