# Supplementary material for: Regime shift detection and neurocomputational substrates for under and overreactions to change
Source: eLife. 2026 May 11;14:RP104684. doi: 10.7554/eLife.104684 (PMC13160555; doi:10.7554/eLife.104684)
Supplement: Supplementary file 12. — Cluster-level inference using Gaussian random field theory (familywise error corrected at p<.05\begin{document}$p{< }.05$\end{document} with a cluster-forming threshold z>3.1\begin{document}$z{> }3.1$\end{document}). [file elife-104684-supp12.docx]

| **Probability estimates** $\boldsymbol{P}_{\boldsymbol{t}}$ **(negative correlation)** | | | | |
| --- | --- | --- | --- | --- |
| **Cluster** | **Hemisphere** | **Cluster size** | **z-max** | **z-max(x,y,z)** |
| Temporal Occipital Fusiform Cortex | L | 27876 | 6.63 | (-24,-54,-22) |
| Supplementary Motor Cortex | R | 1096 | 4.72 | (12,0,46) |
| **Probability estimates** $\boldsymbol{P}_{\boldsymbol{t}}$ **(positive correlation)** | | | | |
| Postcentral Gyrus | L | 705 | 4.73 | (-46,-28,54) |
| **Belief revision** $\boldsymbol{\Delta P}_{\boldsymbol{t}}$ **(positive correlation)** | | | | |
| Frontal Orbital Cortex | L | 8939 | 5.02 | (-24,20,-10) |
| Frontal Orbital Cortex | R | 2244 | 4.7 | (20,6,-14) |
| Lateral Occipital Cortex, superior division | L | 1786 | 4.45 | (-28,-82,34) |
| Postcentral Gyrus | L | 1752 | 4.61 | (-58,-24,38) |
| Postcentral Gyrus | R | 1053 | 4.61 | (60,-18,36) |
| Precentral Gyrus | L | 361 | 3.92 | (-52,6,32) |
