# Supplementary material for: Regime shift detection and neurocomputational substrates for under and overreactions to change
Source: eLife. 2026 May 11;14:RP104684. doi: 10.7554/eLife.104684 (PMC13160555; doi:10.7554/eLife.104684)
Supplement: Supplementary file 13. — Cluster-level inference using Gaussian random field theory (familywise error corrected at p < 0.05 with a cluster-forming threshold z>3.1\begin{document}$z{> }3.1$\end{document}). [file elife-104684-supp13.docx]

| **Intertemporal prior (negative correlation)** | | | | |
| --- | --- | --- | --- | --- |
| **Cluster** | **Hemisphere** | **Cluster size** | **z-max** | $\boldsymbol{z-}\mathbf{max}\boldsymbol{(x,y,z)}$ |
| Temporal Occipital Fusiform Cortex | R | 2101 | 5.3 | (44,-54,-20) |
| Lateral Occipital Cortex, inferior division | L | 976 | 4.49 | (-40,-82,-8) |
| Paracingulate Gyrus | R | 316 | 4.12 | (14,34,30) |
| Right Thalamus | R | 240 | 4.32 | (22,-28,-4) |
| Precuneus Cortex | R | 230 | 4.13 | (6,-82,48) |
| Frontal Pole | R | 186 | 3.73 | (42,44,34) |
| $\mathbf{ln}\left( \boldsymbol{d} \right)$**× signal (positive correlation)** | | | | |
| Supramarginal Gyrus, posterior division | L | 945 | 4.58 | (-50,-42,56) |
| Supramarginal Gyrus, posterior division | R | 923 | 4.44 | (46,-40,48) |
| Middle Frontal Gyrus | R | 785 | 4.19 | (46,12,50) |
| Superior Frontal Gyrus | R | 521 | 4.49 | (6,32,50) |
| Middle Frontal Gyrus | L | 226 | 4.1 | (-46,34,30) |
